# Loss of Stability for Blood Urea Nitrogen in Cattle Serum Samples Stored for 3 and 6 Months at −20°C and −80°C

**DOI:** 10.1111/vcp.70114

**Published:** 2026-05-17

**Authors:** I. Lizarraga, P. Reid, K. Wieliczko, J. Beeler‐Marfisi, F. Castillo‐Alcala

**Affiliations:** ^1^ Bioeconomy Science Institute AgResearch Limited, Grasslands Research Centre Palmerston North New Zealand; ^2^ Department of Pathobiology, Ontario Veterinary College University of Guelph Guelph Ontario Canada; ^3^ Tāwharau Ora – School of Veterinary Science Massey University Palmerston North New Zealand

**Keywords:** agreement analysis, biochemistry, blood urea, bovine, sample stability, storage conditions

## Abstract

**Background:**

Biological samples are stored for delayed or add‐on analyses. However, storage conditions, including time and temperature, can affect the stability of measurands in the samples.

**Objectives:**

To assess the stability of blood urea nitrogen (BUN) in cattle serum samples.

**Methods:**

Blood samples collected from 72 heifers were immediately analyzed for BUN by a commercial laboratory and our in‐house laboratory (optimal conditions, *t*_0_). In‐house, samples were also analyzed after storage at −20°C and −80°C for 3 and 6 months (test samples, *t*_x_). Agreement between laboratories was assessed using Passing‐Bablok and Bland–Altman analyses. The maximum permissible instability (MPI = 4%) was calculated as the sum of in‐house intra‐ and inter‐assay analytical variation. Percentage deviation (PD% = [(*t*_x_–*t*_0_)/*t*_0_] × 100) was estimated for each frozen sample. Stability was assessed by comparing individual PD% against the MPI.

**Results:**

Our in‐house analytical methodology performed within the ASVCP recommended guidelines. BUN (mmol/L) ranged from 1.9 to 5.4 in the commercial laboratory and 1.6 to 6.1 in our in‐house laboratory. There was poor agreement with proportional bias in which the higher the BUN concentration, the greater the difference between laboratories. Storage at −20°C and −80°C yielded PD% between −18.6% and 27.1% after 3 months and −17.5% and 71.6% after 6 months. Regardless of freezing temperature, 54% and 80% of PD% values were greater than the MPI after 3‐ and 6‐month's storage, respectively.

**Conclusions:**

Although cattle serum sample degradation begins by three months storage time, further studies are needed to establish the actual stability limit for BUN.

## 1 Introduction

Urea is the second largest pool of nitrogen (N) in the blood after N in circulating proteins. Urea is produced in the liver from the degradation products of N‐containing molecules. Therefore, urea production changes in parallel to the degradation of dietary and endogenous proteins [1]. In ruminants, dietary protein is degraded into peptides, amino acids, and ammonia, and the latter is used by ruminal bacteria to synthesize microbial protein. Ammonia not utilized by rumen microbes is absorbed through the rumen wall into blood, and is then catabolized into urea in the liver. Urea is subsequently released into the circulation and excreted mainly by the kidneys in urine [2]. However, urea produced in the liver is not only a waste product of N metabolism. Blood urea is transported through the rumen epithelium, and ruminal bacteria can use urea N for amino acid and protein biosynthesis after hydrolyzing it to carbon dioxide and ammonia by ureases [3]. This metabolic machinery also allows the inclusion of urea in the feed for ruminants with a functional rumen, which provides non‐protein N for bacterial protein synthesis in partial replacement of other dietary protein sources [4].

About 25% of dietary N is utilized by dairy cattle [5], and this low N use efficiency results in N losses that contribute to major environmental problems. For instance, N leached from pastureland enters waterways and causes freshwater eutrophication, which alters biodiversity [6]. Also, because protein is generally the most expensive component of cattle diets, inefficient N use represents an economic loss [5]. Additionally, the production of protein feeds in some parts of the world also threatens ecosystems due to deforestation, changes in land use, and increased resource consumption [7]. Therefore, legislative, economic, environmental, and societal pressures are focusing attention on both reducing N inputs into and N losses from animal agriculture [7].

In cattle, blood urea nitrogen (BUN) concentration has been used as an indicator of the need for protein supplementation [8] and a predictor of urinary N excretion [9]. The latter has led investigators to use BUN as a proxy measure of urinary N excretion [5, 10]. Although BUN concentration is similar in fresh cattle serum and plasma samples [11], it is also common for samples to be stored frozen for months prior to assay. For instance, retrospective studies may rely on data obtained from samples that have been stored frozen for long periods [12]. Similarly, prospective studies may require multiple experimental time points, which often results in samples being batch analyzed and thus subjected to different storage times [13]. In addition to storage times, storage temperature may affect the stability of some analytes, but it has been reported that freezing at −70°C results in less analyte variability than at −20°C [14, 15]. Therefore, both storage time and temperature are preanalytical factors to consider when designing an experiment that relies on frozen samples for analysis.

The effects of storage time and temperature on the stability of BUN have been studied in humans [16, 17, 18, 19, 20, 21], and laboratory [15], companion [14, 22, 23] and production animals [24, 25, 26, 27] with mixed results. These conflicting results could be attributed to species differences but also, at least partially, to the wide range of storage temperatures (i.e., room temperature to −160°C) and times (i.e., 1 day to 6 years). For instance, BUN in rat serum increased by 6.5% and decreased by 0.3% after being stored for 360 days at −20°C and −70°C, respectively [15]. Only one published study in cattle reported that BUN was stable for at least 6 days when serum samples were stored at 4°C and at least 6 months when stored at −20°C [25]. Therefore, the paucity of information about storage conditions and the stability of BUN in cattle blood samples led us to assess the stability of this analyte in cattle serum samples stored for up to 6 months at two temperatures. We hypothesized that BUN concentrations would decrease over time in samples stored at −20°C but would remain stable in samples stored at −80°C.

## 2 Materials and Methods

### 2.1 Animals

Seventy‐two Friesian × Hereford heifers with a mean ± SD age of 687 ± 9.9 days and liveweight of 531.2 ± 35.5 kg were used in this experiment. The animals were enrolled in a study assessing the effect of weaning age on growth performance and methane production [28]. The animals were raised on mixed perennial ryegrass and white clover pastures at. As part of routine care, each heifer had regular parasite drenching. During scheduled sampling in the weaning trial, additional blood samples were collected from 36 animals on 15 February 2024 and 36 animals on 12 March 2024, which coincided with the end of summer in the southern hemisphere. Three weeks before the first sampling, half of the animals continued grazing on mixed perennial ryegrass and white clover pastures and the other half were on chicory. The animals were then switched to the other pasture following a crossover design. All animal manipulations were approved by the AgResearch Animal Ethics Committee (AE 2202).

### 2.2 Blood Collection and Processing

Blood samples were collected between 9:00 AM and 12:00 PM by percutaneous venipuncture performed by experienced staff. A 20G × 1 blood collection needle (BD Vacutainer PrecisionGlide 360 214, Beckton, Dickson and Company, Roborough, Plymouth, UK) attached to a vacutainer needle holder (BD Vacutainer Needle Holder Yellow 364 879, Beckton, Dickson and Company) was inserted into an external jugular vein. From each animal, blood was collected into two 10 mL blood collection tubes (BD Vacutainer Clot Activator Tube Plus Blood Collection Tubes 367 895, Beckton, Dickson and Company). Each tube was filled until the vacuum was exhausted and blood ceased to flow. Then, the tubes were immediately dislodged from the needle, gently inverted 5 times, labeled for identification, placed in a tube rack in an upright position, and left to clot for 30 to 210 min at ambient temperature, which fluctuated between 19°C and 21°C.

Within 30 min of collecting the last blood sample each day, samples were submitted for processing and analysis. One set of samples was submitted to a commercial laboratory (Awanui Veterinary, Palmerston North, New Zealand). This is an International Accreditation New Zealand accredited testing laboratory that meets the requirements of ISO/IEC 17025 General requirements for the competence of testing and calibration laboratories. Upon receipt, the laboratory processed the samples for determination of BUN in serum, based on proprietary standard operating protocols.

The other set of samples was transported to our in‐house laboratory. Samples were immediately centrifuged at 2100 × g for 20 min at room temperature (20°C–22°C; Sigma 3‐18 K, Sigma Laboratory Centrifuges, Osterode am Harz, Germany) and five ~0.75 mL serum aliquots were transferred into 1.5 mL polypropylene vials (Safe‐Lock tubes 0030120086, Eppendorf, Hamburg, Germany), which were tightly sealed. Four sets of vials were immediately stored; two sets at −20°C ± 2°C and two sets at −80°C ± 4°C. The freezers were not self‐defrosting and were on automatic emergency power back up and monitored for temperature using thermometers and high temperature alarms. The remaining aliquot set was immediately used to determine BUN in our in‐house laboratory. Temperature notation was consistently expressed in numerical form followed by the ^o^C symbol and the abbreviated scale for Celsius (C).

### 2.3 Determination of BUN

In the commercial laboratory, a kinematic test with urease and glutamate dehydrogenase (Ureal, Roche Diagnostics GmbH, Mannheim, Germany) was used to quantify BUN on a cobas 6000 analyzer with the c501 module (Roche Diagnostics GmbH). The analytical test has a reported measuring range of 0.5–40 mmol/L with coefficients of variation for accuracy and precision of 1% and 1.2%, respectively, using quality control material (PreciNorm U Plus, Roche Diagnostics GmbH).

In our in‐house laboratory, BUN was determined also using an enzymatic (urease and glutamate dehydrogenase) kinematic method but from a different provider (UR446, Randox Laboratories Ltd., Dungloe, Donegal, Ireland). All samples were run in duplicate in flat bottom, 96‐well plates (Serological plate SLP‐000‐096, Guangzhou Jet Bio‐Filtration Co. Ltd., Guangzhou, China) and read in a UV–Vis microplate spectrophotometer at 340 nm UV light at 37°C (Spectramax 250, Molecular Devices Corporation, Sunnyvale, CA, USA). Linear regression analysis of the internal standard versus absorbance enabled the determination of BUN concentration. The method was linear (*r* ^2^ ≥ 0.9722) between 2.13 and 12.82 mmol/L and the manufacturer specifies that it is linear up to 50 mmol/L. Each sample and standard concentration were measured in duplicates, and the average measurement was considered the final value.

The performance of the analytical methodology used for in‐house determination of BUN was evaluated according to guidelines from the American Society for Veterinary Clinical Pathology (ASVCP; [29]). Quality control material (urea calibration standard UR446, Randox Laboratories Ltd.; lot 3431UR) was measured daily for 7 days to determine inter‐assay bias (inaccuracy) and imprecision (random error). Quality control material was also measured for 20 replicates to determine intra‐assay bias and imprecision.

### 2.4 Stability Test

The stability test was carried out in‐house. After being stored for 3 months, one set of serum aliquots frozen at −20°C and another set frozen at −80°C were passively warmed to room temperature for 20 min and BUN was measured using the in‐house method described above. The same was done with the remaining two aliquot sets (i.e., one set frozen at −20°C and the other set at −80°C) after being stored for 6 months. Each aliquot underwent only a single freeze–thaw cycle prior to BUN measurement.

The maximum permissible instability (MPI), defined as the maximum allowable deviation for BUN concentration from serum samples processed under optimal conditions on day 0 in our in‐house laboratory, was calculated as the sum of intra‐ and inter‐assay analytical variation (*CV*_*a*_) using the formula MPI = *CV*_*a‐intra*_ + *CV*_*a‐inter*_ and expressed as percentage value. Percentage deviation (PD%) was estimated for each of the frozen aliquots as the difference between BUN concentration determined under optimal conditions (i.e., day 0; baseline sample or *t*_0_, average of replicate measurements) and BUN concentration determined in aliquots stored for 3 months at −20°C, 3 months at −80°C, 6 months at −20°C, and 6 months at −80°C (i.e., test samples or *t*_x_, average of replicate measurements). The formula PD% = [(*t*_x_—*t*_0_)/*t*_0_] × 100 was used for this purpose [30].

### 2.5 Statistical Analysis

The number of animals included in the study (*n* = 72) is larger than the minimum recommended sample size for a comprehensive stability test (*n* = 10; [30]). In‐house analytical performance was determined by computing the observed total error (*TE*_*obs*_). The mean (*x̄*) and standard deviation (SD) were used to calculate the coefficient of variation [*CV*; *CV(%)* = (SD/*x̄*) × 100] and bias [bias(%) = (*x̄*_manufacturer_—*x̄*_measured_/ *x̄*_manufacturer_) × 100]. Bias and *CV* were then used to estimate *TE*_*obs*_ [*TE*_*obs*_ (%) = bias + 2*CV*]. An inter‐assay TE_obs_ less than 12%, the recommended total allowable error (*TE*_*a*_) for BUN, indicates that the instrument is functioning within ASVCP guidelines [29].

Agreement for the determination of BUN between the accredited commercial laboratory and our in‐house laboratory was established using Passing–Bablok regression and Bland–Altman plot analyses. Agreement was considered good when the 95% CIs for the slope and intercept from the Passing–Bablok regression included the value 1 and 0, respectively [31]. Limits of agreement in the Bland–Altman plot analysis were determined using the mean and the standard deviation of the differences between the two measurements. If the line of equality was not within the 95% CI interval of the mean, there was a significant systematic difference between methods [32]. The Passing–Bablok regression and Bland–Altman plot analyses were computed using SigmaPlot v14.0 (Systat Software Inc., San Jose, CA, USA) and the web‐based, open‐source BA‐plotteR (https://huygens.science.uva.nl/BA‐plotteR/; [33]), respectively.

Stability of BUN under the evaluated storage conditions was assessed by comparing individual PD% values and the calculated MPI. A PD% smaller than the MPI for all studied subjects indicated no significant loss of stability within the storage conditions. A PD% equal to or greater than the MPI in some of the studied subjects indicated a significant loss of stability within the storage conditions [30]. The distribution of the PD% values was analyzed using the Shapiro–Wilk test and PD% values were expressed as range, median, and interquartile range. For completeness, absolute concentration differences in BUN were also reported (SigmaPlot v14.0).

## 3 Results

All samples were grossly negative for hemolysis, lipemia, and icterus, and the commercial laboratory did not report the presence of any of these potential interferents either. The inter‐assay performance values for our in‐house analytical methodology for BUN determination were mean = 12.77 mmol/L, SD = 0.14 mmol/L, *CV*_*a*_ = 1.08%, *n* = 7, bias = 0.41%, and observed total error (*TE*_*obs*_) = 2.57%. Corresponding intra‐assay values were 13.04 mmol/L, 0.38 mmol/L, 2.89%, 20, −1.72%, and 7.49%.

The BUN values determined in our in‐house laboratory ranged from 1.6 to 6.1 mmol/L (mean ± SD = 3.36 ± 0.85 mmol/L) and those in the commercial laboratory ranged from 1.9 to 5.4 mmol/L (mean ± SD = 3.22 ± 0.73 mmol/L). The Passing–Bablok and the Bland–Altman plots and their statistical values for BUN are depicted in Figure 1 and Table 1, respectively. The Passing–Bablok regression analysis showed poor agreement between the commercial laboratory and our in‐house laboratory results. The intercept was computed at 0.4328 (95% CI, 0.2523 to 0.6134) and the slope at 0.8287 (95% CI, 0.7766 to 0.8808) (Figure 1A). The Bland–Altman plot analysis confirmed poor agreement with the commercial laboratory providing BUN results with a mean difference of −0.1431 mmol/L (95% CI, −0.1988 to −0.0873) compared to our in‐house laboratory. The 95% lower limit of agreement was −0.6046 mmol/L (95% CIs, −0.6998 to −0.5099) and the 95% upper limit of agreement was 0.3185 mmol/L (95% CI, 0.2232 to 0.4137) (Figure 1B). The intercept of the difference was 0.372 mmol/L (95% CI, 0.1643 to 0.5797) and the slope of the difference was −0.1565 (95% CI, −0.2179 to −0.0951). This suggested proportional bias and regression‐based limits of agreement were plotted (Figure 1C). The data were also analyzed by sampling date, which also identified poor agreement between laboratories (Table 1 and Figures S1 and S2).

**FIGURE 1:**
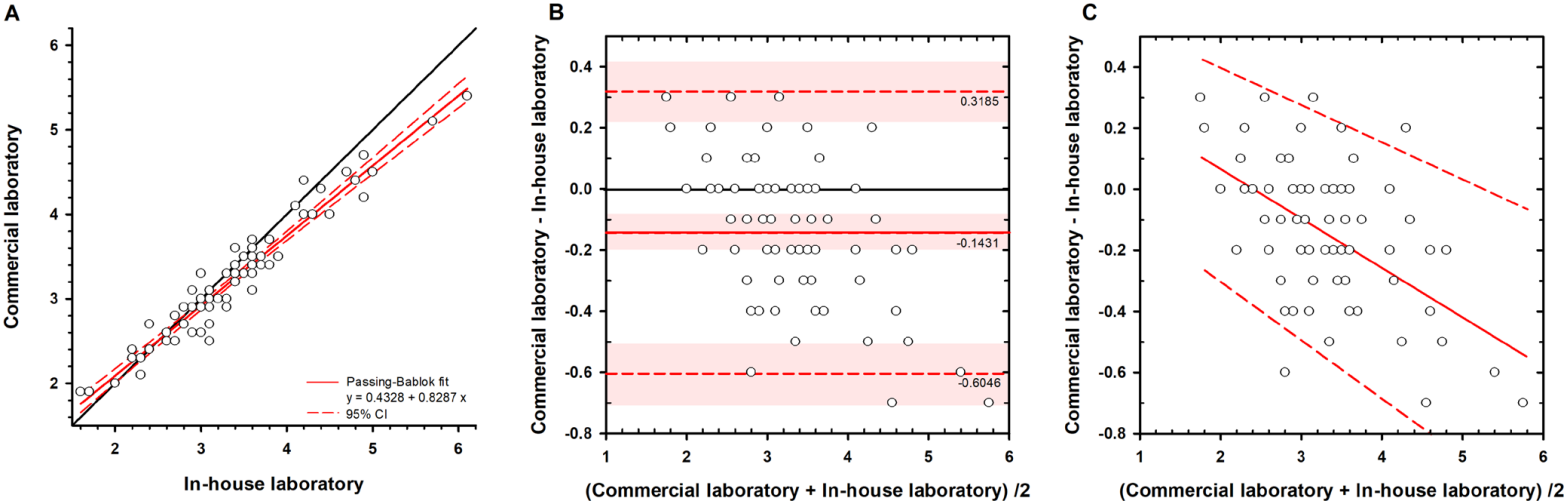
Passing–Bablok (A) and Bland–Altman (B and C) plots showing the agreement between a commercial laboratory and our in‐house laboratory for the determination of blood urea nitrogen (BUN) in cattle serum samples (*n* = 72). The Passing–Bablok plot (A) contains scatter data (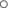) with regression line (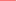) and 95% confidence intervals (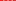) in red and identity line (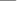) in black. The Bland–Altman plots (B and C) contain scatter differences between laboratories (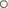) with means (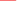) and limits of agreement (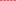) in red. The Bland–Altman plot B also contains 95% confidence intervals for the mean and limits of agreement (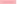) in shaded red, and the identity line (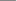) in black.

**TABLE 1:** Passing–Bablok regression and Bland–Altman plot statistics for assessment of agreement between the commercial laboratory and our in‐house laboratory for the determination of blood urea nitrogen (BUN) in cattle serum samples.

Samples	Passing–Bablok		Bland–Altman					Agreement
	Intercept (95% CI)	Slope (95% CI)	Mean difference (95% CI)	95% Lower LoA (95% CI)	95% Upper LoA (95% CI)	Intercept (95% CI)	Slope (95% CI)	
1–72	0.4328 (0.2523 to 0.6134)	0.8287 (0.7766 to 0.8808)	−0.1431 (−0.1988 to −0.0873)	−0.6046 (−0.6998 to −0.5099)	0.3185 (0.2232 to 0.4137)	0.372 (0.1643 to 0.5797)	−0.1565 (−0.2179 to −0.0951)	Poor
1–36	0.43 (0.2027 to 0.6574)	0.8301 (0.7692 to 0.8910)	−0.1806 (−0.2657 to −0.0954)	−0.6671 (−0.8127 to −0.5214)	0.3059 (0.1603 to 0.4516)	0.4007 (0.1395 to 0.662)	−0.1659 (−0.238 to −0.0938)	Poor
37–72	0.4558 (0.069 to 0.8426)	0.8207 (0.699 to 0.9424)	−0.1056 (−0.1807 to −0.0304)	−0.5348 (−0.6633 to −0.4063)	0.3237 (0.1952 to 0.4522)	0.2608 (−0.1811 to 0.7027)	−0.119 (−0.2607 to 0.0226)	Poor

*Note:* Samples 1–36 were collected on 15 February 2024 and samples 37–72 on 12 March 2024.

Graphical representation of individual BUN data points, denoted as PD%, revealed different patterns between samples collected on the two sampling days (Figure 2). Samples 1–36, collected on 15 February 2024, seemed relatively more stable than samples 37–72, collected on 12 March 2024, irrespective of whether samples were then stored at −20°C or −80°C (Figures S3 and S4). Overall, samples stored for 3 months appeared more stable than those stored for 6 months irrespective of freezing temperature (−20°C or −80°C). The PD% data followed a normal distribution (W‐Statistic ≥ 0.969; *p* ≥ 0.07) and PD% values ranged from −18.7% to 71.6% regardless of freezing conditions. In contrast, absolute concentration differences in BUN data did not follow a normal distribution (W‐Statistic ≥ 0.947; *p* ≤ 0.01, expect at 3 months and −80°C, *p* = 0.772) and values ranged from −3.2 mmol/L to 1 mmol/L. The complete descriptive statistics for PD% and absolute concentration differences in BUN data are presented in Table 2.

**FIGURE 2:**
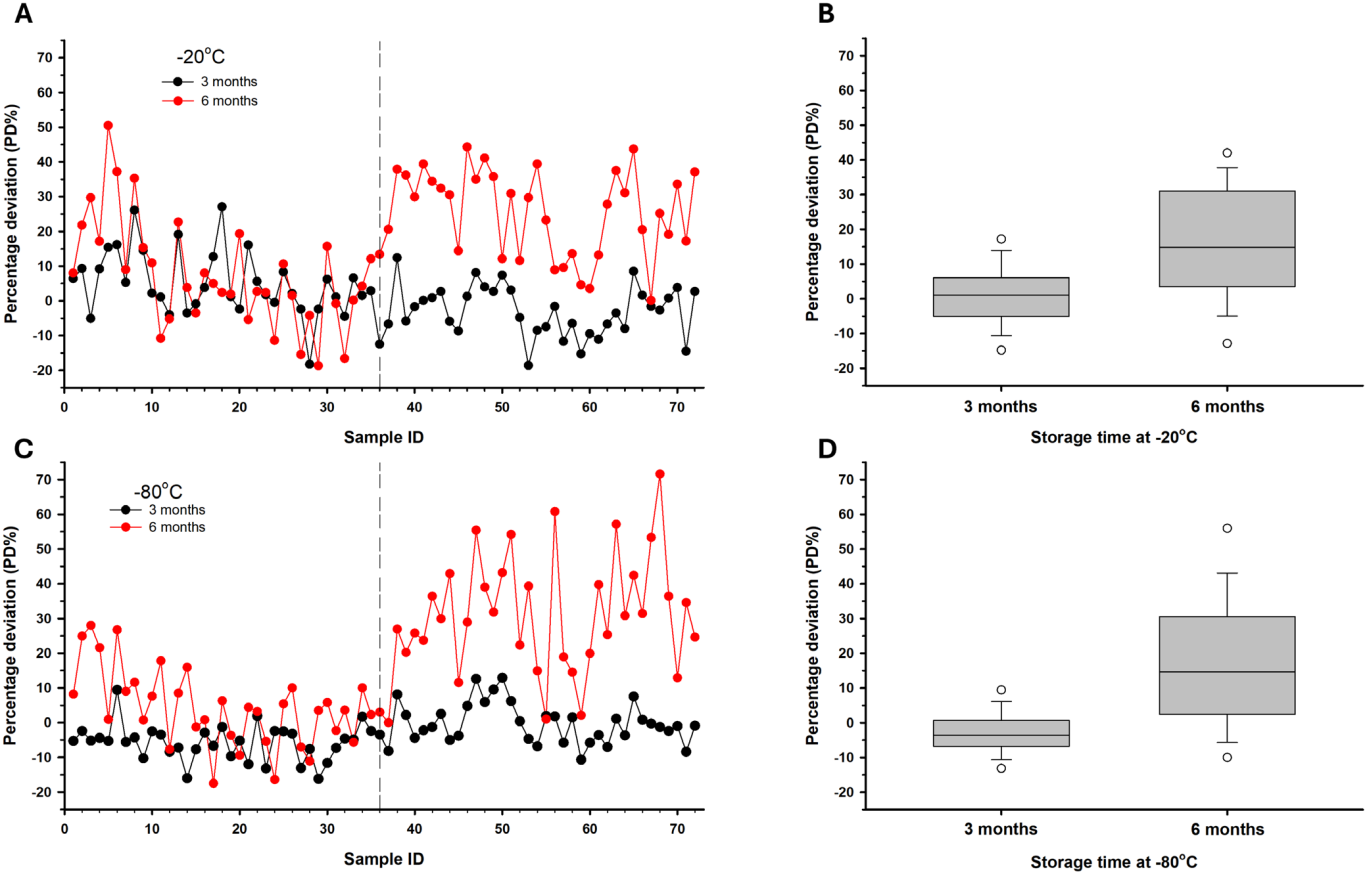
Individual blood urea nitrogen (BUN) percentage deviation (PD%) values in cattle serum samples stored for 3 (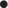) and 6 (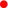) months at −20°C (A) and −80°C (C). The vertical dotted line separates samples by sampling date: Samples 1–36 were collected on 15 February 2024 and samples 37–72 on 12 March 2024. The box and whisker plots show BUN PD% values from samples stored for 3 (left columns) and 6 (right columns) months at −20°C (B) and −80°C (D). The interquartile range contains the middle 50% of PD% values (between the 25th and 75th percentiles) and the median (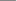). The upper and lower whiskers represent PD% values in the lower 25th and the upper 25th percentiles. The open circles (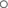) represent the minimum and maximum PD% values, excluding outliers.

**TABLE 2:** Descriptive statistics and data distribution analysis for percentage deviation (PD%) and absolute concentration differences in blood urea nitrogen (BUN, mmol/L) from serum samples stored for 3 or 6 months at −20°C and −80°C (*n* = 72).

Storage time (months)	Storage temperature (°C)	Minimum	Maximum	Median	IQR range	W‐statistics	*p*
		(% or mmol/L)					
PD%							
3	−20	−18.6	27.1	1.1	10.9	0.978	0.248
3	−80	−16.2	12.9	−3.6	7.4	0.975	0.165
6	−20	−18.7	50.5	14.9	27.3	0.973	0.131
6	−80	−17.5	71.6	14.7	27.7	0.969	0.07
Absolute concentration differences (mmol/L)							
3	−20	−1.1	0.9	0	0.4	0.954	0.01
3	−80	−1.7	1	−0.5	0.8	0.989	0.772
6	−20	−0.4	0.8	0.1	0.2	0.947	0.004
6	−80	−3.2	0.8	−0.5	0.8	0.952	0.008

The established MPI was 4% (i.e., *CV*_*a‐intra*_ + *CV*_*a‐inter*_ = 2.89% + 1.08% = 3.97%) and there was a significant loss of BUN stability within the storage conditions evaluated. Values for PD% were larger than the established MPI in 54.2% and 58.3% of the samples stored for 3 months at −20°C and −80°C, respectively. Also, most samples stored for 6 months at −20°C (84.7%) and −80°C (80.6%) yielded larger PD% values than the established MPI.

## 4 Discussion

Biobanks can be a useful source of information; however, it is important to establish how sample handling, processing, and storage may alter the suitability of samples to test certain hypotheses. Using an in‐house methodology to determine BUN concentrations in cattle serum samples, we demonstrated that freezing this matrix for 3 or 6 months at −20°C and −80°C is not appropriate to preserve the stability of this analyte. Loss of BUN stability was detected in 54% to almost 85% of the samples studied under the specified storage conditions (Figure 2).

The analytical method used in our in‐house laboratory was considered suitable to determine BUN accurately and precisely. This is because the estimated *TE*_*obs*_ was smaller than the 12% TE_a_ recommended by the ASVCP [29]. Although *TE*_*a*_ can also be used as a measure of agreement or comparability of results from different laboratories [29], the degree of agreement for BUN concentrations between our in‐house laboratory and the commercial laboratory was assessed using robust statistical approaches [31, 32]. Poor agreement between laboratories was confirmed by the Passing–Bablok regression statistics because they did not include the value 1 in the 95% CI for the slope nor 0 in the 95% CI for the intercept [31]. Furthermore, the Bland–Altman plot statistics showed that, on average, our in‐house analytical methodology measured 0.14 mmol/L lower than the commercial laboratory. This negative bias was mainly observed in samples with BUN values over 3 mmol/L, while for lower concentrations, data were closer to each other. BUN concentrations measured by the commercial laboratory ranged from 0.6 mmol/L below or 0.32 mmol/L above our in‐house laboratory. Although a significant constant over‐estimation in BUN concentrations by our in‐house laboratory may be suggested by the line of equality not laying within the 95% CI of the mean difference [32], 95% CI values for the intercept and the slope suggested proportional bias. Thus, the greater the BUN concentration, the larger the difference in results between laboratories, which was represented as a slope of the regression line of the graph different from zero (Figure 1C; [33]).

Because of different analytical methods, it is generally expected that in‐house tests and reference laboratory tests will produce different results for identical samples [34], which can also happen between runs on the same in‐house analyzer [35]. In addition to analytical factors, pre‐ and post‐analytical factors should also be considered when interpreting clinical pathology results [25, 36, 37]. In the current study, pre‐analytical factors were similar until the samples were delivered to the laboratories, where post‐analytical factors were minimized by rigorous quality control. Our findings reiterate that results obtained using in‐house methods compared to reference methods are generally not interchangeable unless appropriate statistical assessment has determined agreement between them. Despite differences between laboratories, the BUN concentrations determined in both laboratories were within recommended reference intervals for the species (i.e., 1.9 to 8 mmol/L; [38]) and were trending in the same direction. Nevertheless, the same laboratory should always be used when comparing results from the same individual or during a research trial. This would reduce subtle variations in results that may not be immediately noticeable yet may influence clinical or research outcomes.

The performance of our in‐house analytical methodology complied with ASVCP recommended guidelines to determine BUN [29]. Therefore, it was deemed both reliable and appropriate to assess in‐house the stability of BUN at predetermined storage conditions. Serum was chosen because it is the preferred matrix at our institution for delayed analysis or add‐on tests. It is usually aliquoted and stored frozen at −20°C or −80°C for several months before batch analysis. Our data showed that, under the conditions of our study, freezing cattle serum samples at −20°C or −80°C for 3 and 6 months affected the stability of BUN. Thus, regardless of freezing temperature, loss of stability was observed as early as 3 months storage time, but it is unknown if stability is retained before that.

The concentration of BUN in human serum has been reported as stable for up to 3 days when samples were stored at 4°C or room temperature (23°C ± 1°C) [16], but increased significantly after keeping serum samples uncapped at room temperature for 16 h and then storing them at 4°C for 4 days [19] or after storing samples at −20°C for more than 5 and up to 10 days [20]. A 6.5% to 13.3% increase in BUN concentration was found in serum samples stored at −20°C for 1 to 3 months [18]. Storing serum samples from pediatric patients at −80°C for up to 13 months also yielded a 6.7% change in BUN concentration; however, a decrease of 15.6% was observed in samples from 1‐ to 30‐day‐olds [17]. Although storage of serum samples in vapor‐phase liquid nitrogen (−180°C) for 6 years did not affect BUN concentration [21], this condition was not evaluated in the current study.

The stability of BUN has also been evaluated in laboratory and domestic animals. For instance, BUN concentration in rat serum samples increased by 0.2% when stored at 4°C for 7 days and by 6.5% when stored for up to 1 year at −20°C, and decreased by 0.3% when stored for up to 1 year at −70°C [15]. In dogs, BUN increased by 10.9% in plasma samples stored at −20°C for 3 days [23] and by 3.4% in serum samples stored at −20°C for 8 months [14]. An increase in BUN concentration of 3.9% in cats and 3.6% in horses was observed after storing plasma samples at −20°C for 7 days [22]. A 4.3% increase in BUN concentration was reported in donkey serum samples stored at −80°C for 1 year [24]. In contrast, a statistically significant decrease in BUN concentration was reported when serum samples from goats, horses, and oxen were stored for 1 and 2 months at −20°C, yet BUN remained stable in sheep serum samples stored under the same conditions [26, 27]. A study in lactating dairy cows reported that BUN in serum samples was stable for at least 6 days when samples were transported on dry ice, processed within 4 h of sampling, and stored at 4°C, and BUN was stable for at least 6 months when samples were stored at −20°C [25]. This contrasts with the loss of BUN stability reported in our study after storing cattle serum samples for 3 and 6 months at −20°C and −80°C. Additionally, preanalytical factors such as prolonged transport (4–8 h), transport at ambient temperature, small sample volume, or hemolysis have been implicated in reduced BUN stability by 2–4 days when cattle serum samples were stored at 4°C and by 1 month when stored at −20°C [25]. Thus, it could be possible that transporting cattle serum samples at ambient temperature may have influenced the loss of BUN stability in our study.

It is worth noting that direct comparisons of the above‐mentioned BUN stabilities may not be appropriate. Differences in stability may arise from pre‐analytical factors, including storage conditions, but they may also be attributed to post‐analytical factors such as different methods to define significant change. Using inferential statistical tests for paired observations would determine statistically significant change [15, 16, 20, 26, 27], but it would not consider the *CV*_*a*_ and may thus produce misleading results. More appropriate methods to determine sample stability include critical change value [CCV, a.k.a. reference change value (RCV)] [25], TE_a_ [29], and MPI [30]. We used the latter method because samples were collected only once from each heifer and thus we did not include biological variation in our model. An MPI of 4% was established for BUN based on *CV*_*a*_ in the current study and it was similar to that in a study in lactating dairy cows (3%; [25]). The computed intra‐ and inter‐assay *CV*_*a*_ were similar to those reported by others using the same BUN analytical methods but quantified on a different analyzer (1.62%; [39]) or using the same analytical method from a different provider on a different analyzer (2.3%; [38]). Nevertheless, extrapolation of results from the current study to other laboratories may be challenging, which highlights the need for standard guidelines for the execution of stability tests [30].

The stability of BUN in serum samples could have been affected by the elapsed time between sample collection and centrifugation, storage time in the primary container at room temperature after centrifugation, and storage conditions in the secondary container [30]. Although blood samples were left to clot from 1 to 4 h before centrifugation, this was a much shorter time than that experienced by the majority of large animal samples submitted to veterinary diagnostic laboratories. Additionally, because the actual collection time or order of collection for each sample was not systematically recorded, the effect of clotting duration on baseline BUN concentrations and subsequent PD% values could not be assessed. Serum was harvested from the primary container (i.e., Vacutainer tube) within 20 min of blood being centrifuged and then immediately transferred to a secondary container (i.e., Eppendorf tube). The secondary container was tightly sealed to prevent evaporative concentration effects and then placed in a freezer. Interestingly, prolonged transport (4–8 h), hemolysis, low volume sample, and transport at ambient temperature have been identified as affecting the stability of BUN in cattle serum samples [25]. The later study, however, did not specify the degree of hemolysis in their samples and others have demonstrated that up to 10% hemolysis does not affect BUN concentration in cattle serum samples [40]; we did not observe gross hemolysis in our samples. Importantly, all these three studies used an enzymatic (urease/glutamate dehydrogenase) kinematic method to determine BUN. Although it is not completely clear whether PD% changes resulted from pre‐analytical, analytical, and/or post‐analytical error, transporting the samples at ambient temperature may have contributed to the changes observed in our study [25]. In addition, or alternatively, the reported instability may have been due to post‐freezing changes, including crystallization effects, protein unfolding, enzymatic degradation, or other matrix alterations [30, 41]. However, the underlying mechanisms causing BUN instability were not investigated.

Samples with concentrations near clinical decision limits have been recommended for stability studies [30]. Although BUN concentrations under optimal conditions on day 0 were towards the lower end of the reference interval for the analyte and species [38], about 80% of samples were between concentrations of 2 to 4 mmol/L. Whether different results to the ones reported here may be obtained using samples with higher BUN concentrations needs to be determined. All the heifers in the current study were apparently healthy and kept under the same environmental conditions, feeding regimes, and nursing. Recruiting animals with expected higher BUN concentrations would have required controlled interventions such as changes in the diet, feeding time, water intake, and the productive physiological status, all of which are known to affect BUN concentrations in cattle [42, 43]. Because we used animals enrolled in another study, we were not able to modify any of their rearing or nutritional conditions. Alternatively, including animals with disease conditions such as diarrhea or malnourishment [5, 13, 44, 45] or specific genetics [10] may have assisted with procuring samples with more extreme BUN concentrations.

Other limitations of the study include the analysis of only two time points (i.e., 3 and 6 months) which precluded us from establishing the period in which BUN does not exceed the MPI (a.k.a. stability limit; [30]). Further studies analyzing samples at earlier time points (e.g., 1 week, 1 month, or 2 months) would be required to determine a stability limit for BUN in samples stored at −20°C and −80°C. Storage at ultra‐low temperatures (e.g., liquid nitrogen, cryopreservation in freezers at −140°C or lower) would not be practical in our institution but could be considered by others. Repeated sample freezing and thawing was not studied here but is known to affect the stability of other blood analytes [18, 23]. Similarly, the stability of analytes can differ according to the specimen sample analyzed (i.e., serum vs. plasma) [46, 47] and different analytes have different stability characteristics [25]. Thus, various freezing temperatures, freezing/thawing cycles, biological matrices, and measurands of clinical/research relevance should be considered in further studies. All this would help to validate preanalytical processes and ultimately provide a more comprehensive view of the value of bovine biobanks as reliable sources of information.

The performance of our in‐house analytical methodology was satisfactory and functioned within ASVCP recommended guidelines to determine BUN. As expected, it produced dissimilar results than those produced by an accredited commercial laboratory as assessed by Passing–Bablok regression and Bland–Altman plot analyses, although important trends were maintained. Frozen serum samples stored for 3 and 6 months at −20°C and −80°C yielded PD% values larger than the established MPI of 4% indicating loss of stability. However, the stability limit, the underlying mechanisms inducing instability, and the generalizability to other laboratories or freezer systems remain uncertain. Until further formal studies establish the stability limit, it is recommended to analyze fresh bovine blood serum samples under optimal conditions if BUN is the analyte of interest.

## Conflicts of Interest

The authors declare no conflicts of interest.

## Supporting information


**Figure S1:** Passing–Bablok (A) and Bland–Altman (B and C) plots showing the agreement between the commercial laboratory and our in‐house laboratory for the determination of blood urea nitrogen (BUN) in cattle serum samples 1–36 collected on 15 February 2024. The Passing–Bablok plot (A) contains scatter data (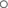) with regression line (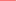) and 95% confidence intervals (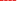) in red and identity line (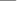) in black. The Bland–Altman plots (B and C) contain scatter differences between laboratories (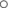) with means (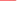) and limits of agreement (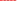) in red. The Bland–Altman plot B also contains 95% confidence intervals for the mean and limits of agreement (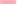) in shaded red, and the identity line (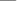) in black.
**Figure S2:** Passing–Bablok (A) and Bland–Altman (B and C) plots showing the agreement between the commercial laboratory and our in‐house laboratory for the determination of blood urea nitrogen (BUN) in cattle serum samples 37–72 collected on 12 March 2024. The Passing–Bablok plot (A) contains scatter data (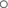) with regression line (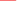) and 95% confidence intervals (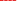) in red and identity line (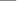) in black. The Bland–Altman plots (B and C) contain scatter differences between laboratories (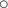) with means (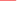) and limits of agreement (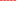) in red. The Bland–Altman plot B also contains 95% confidence intervals for the mean and limits of agreement (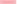) in shaded red, and the identity line (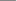) in black.
**Figure S3:** Individual blood urea nitrogen (BUN) percentage deviation (PD%) values in cattle serum samples 1–36 collected on 15 February 2024 and stored for 3 (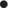) and 6 (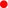) months at −20°C (A) and −80°C (C). The box and whisker plots show BUN PD% values from samples stored for 3 (left columns) and 6 (right columns) months at −20°C (B) and −80°C (D). The interquartile range contains the middle 50% of PD% values (between the 25th and 75th percentiles) and the median (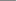). The upper and lower whiskers represent PD% values in the lower 25th and the upper 25th percentiles. The open circles (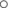) represent the minimum and maximum PD% values, excluding outliers.
**Figure S4:** Individual blood urea nitrogen (BUN) percentage deviation (PD%) values in cattle serum samples 37–72 collected on 12 March 2024 and stored for 3 (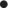) and 6 (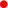) months at −20°C (A) and −80°C (C). The box and whisker plots show BUN PD% values from samples stored for 3 (left columns) and 6 (right columns) months at −20°C (B) and −80°C (D). The interquartile range contains the middle 50% of PD% values (between the 25th and 75th percentiles) and the median (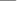). The upper and lower whiskers represent PD% values in the lower 25th and the upper 25th percentiles. The open circles (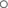) represent the minimum and maximum PD% values, excluding outliers.
