# Supplementary material for: Loss of Stability for Blood Urea Nitrogen in Cattle Serum Samples Stored for 3 and 6 Months at −20°C and −80°C
Source: Vet Clin Pathol. 2026 May 17;55(2):143–51. doi: 10.1111/vcp.70114 (PMC13686598; doi:10.1111/vcp.70114)
Supplement: Supplementary file 1 [file VCP-55-143-s001.docx]

**Supplementary material**

**Loss of stability for blood urea nitrogen in cattle serum samples stored for 3 and 6 months at -20^o^ and -80^o^C**

**Figure S1.** Passing-Bablok (**A**) and Bland-Altman (**B** and **C**) plots showing the agreement between the commercial laboratory and our in-house laboratory for the determination of blood urea nitrogen (BUN) in cattle serum samples 1-36 collected on 15 February 2024. The Passing-Bablok plot (**A**) contains scatter data (○) with regression line (**^_____^**) and 95% confidence intervals (----) in red and identity line (**^_____^**) in black. The Bland-Altman plots (**B** and **C**) contain scatter differences between laboratories (○) with means (**^_____^**) and limits of agreement (-----) in red. The Bland-Altman plot **B** also contains 95% confidence intervals for the mean and limits of agreement () in shaded red, and the identity line (**^_____^**) in black.

**Figure S2.** Passing-Bablok (**A**) and Bland-Altman (**B** and **C**) plots showing the agreement between the commercial laboratory and our in-house laboratory for the determination of blood urea nitrogen (BUN) in cattle serum samples 37-72 collected on 12 March 2024. The Passing-Bablok plot (**A**) contains scatter data (○) with regression line (**^_____^**) and 95% confidence intervals (----) in red and identity line (**^_____^**) in black. The Bland-Altman plots (**B** and **C**) contain scatter differences between laboratories (○) with means (**^_____^**) and limits of agreement (-----) in red. The Bland-Altman plot **B** also contains 95% confidence intervals for the mean and limits of agreement () in shaded red, and the identity line (**^_____^**) in black.

**Figure S3.** Individual blood urea nitrogen (BUN) percentage deviation (PD%) values in cattle serum samples 1-36 collected on 15 February 2024 and stored for 3 (●) and 6 (●) months at -20^o^C (**A**) and -80^o^C (**C**). The box and whisker plots show BUN PD% values from samples stored for 3 (left columns) and 6 (right columns) months at -20^o^C (**B**) and -80^o^C (**D**). The interquartile range contains the middle 50% of PD% values (between the 25^th^ and 75^th^ percentiles) and the median (**^_____^**). The upper and lower whiskers represent PD% values in the lower 25^th^ and the upper 25^th^ percentiles. The open circles (○) represent the minimum and maximum PD% values, excluding outliers.

**Figure S4.** Individual blood urea nitrogen (BUN) percentage deviation (PD%) values in cattle serum samples 37-72 collected on 12 March 2024 and stored for 3 (●) and 6 (●) months at -20^o^C (**A**) and -80^o^C (**C**). The box and whisker plots show BUN PD% values from samples stored for 3 (left columns) and 6 (right columns) months at -20^o^C (**B**) and -80^o^C (**D**). The interquartile range contains the middle 50% of PD% values (between the 25^th^ and 75^th^ percentiles) and the median (**^_____^**). The upper and lower whiskers represent PD% values in the lower 25^th^ and the upper 25^th^ percentiles. The open circles (○) represent the minimum and maximum PD% values, excluding outliers.
